# Understanding motives for intravaginal practices amongst Tanzanian and Ugandan women at high risk of HIV infection: The embodiment of social and cultural norms and well-being^☆^

**DOI:** 10.1016/j.socscimed.2013.12.005

**Published:** 2014-02

**Authors:** Shelley Lees, Flavia Zalwango, Bahati Andrew, Judith Vandepitte, Janet Seeley, Richard J. Hayes, Suzanna C. Francis

**Affiliations:** a London School of Hygiene and Tropical Medicine, Keppel Street, London, WC1E 7HT, United Kingdom; b Medical Research Council/Uganda Research Unit on AIDS, P.O. Box 49, Entebbe, Uganda; c Mwanza Intervention Trials Unit, National Institute for Medical Research Mwanza Centre, P.O. Box 11936, Mwanza, Tanzania; d University of East Anglia, Norwich Research Park, Norwich, NR4 7TJ, United Kingdom

**Keywords:** Intravaginal practices, Hygiene, Sexuality, Tanzania, Uganda, HIV

## Abstract

Some types of intravaginal practices (IVP) may increase the risk for HIV acquisition. This is particularly worrisome for populations with dual high prevalence of HIV and IVP. Women involved in transactional sex are at increased risk for HIV infection in sub-Saharan Africa. Social, cultural and economic influences are strong drivers of IVP in this population. To explore this, we carried out a qualitative research study to investigate the drivers and motivations for using IVP within a large observational study of women at high risk of HIV in Tanzania and Uganda from September 2008 to September 2009. Of the 201 women selected, 176 women took part in a semi-structured in-depth interview. Additionally, in Tanzania, eight focus group discussions among study participants and community members were carried out to obtain information on community norms and expectations. IVP were motivated by overlapping concerns with hygiene, morality, sexual pleasure, fertility, relationship security, and economic security. These motives were driven by the need to meet cultural and social expectations of womanhood, and at the same time attend to personal well-being. Among women involved in transactional sex in East Africa, interventions aimed at modifying or eliminating IVP should attend to local cultural and social norms as well as the individual as an agent of change.

## Highlights

• Previous research has shown that some types of intravaginal practice (IVP) may increase HIV risk.
• The motives of women at high risk of HIV to carry out IVP fulfil sociocultural norms of womanhood and well-being.
• Economic factors for women involved in transactional sex are an important motive.
• Interventions aimed at reducing or eliminating IVP should address these issues.

## Introduction

Intravaginal practices (IVP) are a collection of behaviours in which women modify the structure and environment of the vagina, including intravaginal cleansing and insertion of substances in the vagina (e.g. finger washing with soapy water, insertion of pulverised herbs). IVP are highly prevalent amongst women involved in transactional sex in sub-Saharan Africa (Allen et al., 2010; Fonck et al., 2001; Francis et al., 2013; McClelland et al., 2006; Nagot et al., 2007; Watson-Jones et al., 2007), and this population is also at increased risk of HIV infection (Baral et al., 2012; Kapiga et al., 2002; Vallely et al., 2007; Watson-Jones et al., 2007). Over the past 15 years, there has been growing evidence of an association between some types of IVP and HIV acquisition, as well as reproductive tract infections, such as bacterial vaginosis (Low et al., 2011; Martin Hilber, Francis, et al., 2010; Myer et al., 2004). It is presumed that IVP disrupts the vaginal environment, thus increasing susceptibility to infection. Additionally, these practices may interfere with female controlled HIV prevention methods such as microbicides (Van Der Straten, Cheng, Chidanyika, De Bruyn, & Padian, 2010).

Three studies have investigated interventions to reduce or eliminate IVP: two studies in the US aimed to reduce douching; and one study in Kenya investigated the feasibility of intravaginal cleansing cessation (Grimley, Oh, Desmond, Hook, & Vermund, 2005; Klebanoff et al., 2006; Masese et al., 2012). All studies reported a reduction in vaginal cleansing or douching, although it is not known whether this reduction was maintained after the studies ended. The two US studies focused on individual behaviour change and adherence. The study in Kenya focused on individual behaviour change, and individual motivations and social support for IVP cessation.

Several studies exploring IVP in sub-Saharan Africa have revealed broad social, economic and cultural influences on IVP (Allen et al., 2010; Bagnol & Mariano, 2008; Braunstein & van der Wijgert, 2003; Brown & Brown, 2000; Martin Hilber, Francis, et al., 2010; Scorgie et al., 2009). Amongst these studies a complex picture of motivations for IVP has emerged. A meta-ethnography of vaginal practices by Martin Hilber et al. (2012) revealed that IVP are influenced by a multitude of concerns including fertility, health, economic security, the supernatural world, and community or social norms. Following a global review of literature related to vaginal practices, Braunstein and van der Wijgert (2003) suggested that IVP enforce social norms relating to gender, sexuality, the body, and health. Both reviews strongly suggest that IVP are influenced predominantly by social, cultural and gender norms and these are usually instilled in the process of socialisation of young girls into adulthood. In many studies, there is an overarching theme of the ‘preferred vaginal state’, which relates to cleanliness, the right amount of lubrication, and tightness (Allen et al., 2010; Braunstein & van der Wijgert, 2003; Martin Hilber, Francis, et al., 2010; Martin Hilber et al., 2012). In-depth ethnographic work with Tanzanian women who participated in an HIV prevention trial has revealed that this preferred vaginal state draws on moral ideas of the clean and chaste woman (Lees, 2013). IVP may also have economic motivations (Martin Hilber et al., 2009), especially among women who are involved in transactional sex.

In sub-Saharan Africa, transactional sex has been described as a continuum from gift-giving in long-term relationships to formalised commercial sex work (MacPherson et al., 2012). Women involved in transactional sex are expected by male clients to present themselves with the preferred vaginal state (Hull et al., 2011; Lees, 2013; Martin Hilber et al., 2012). This expectation, alongside concerns about HIV infection, may influence the frequency and prevalence of IVP amongst women who are involved in transactional sex. For example, a study in Tanzania found that cleansing was conducted more often before transactional sex than non-transactional sex to ensure cleanliness, and after transactional sex to reduce the risk of HIV infection (Allen et al., 2010). Furthermore, higher frequency of sex amongst women involved in transactional sex has been found to be associated with more frequent intravaginal cleansing and insertion (Francis et al., 2013).

In the light of the potential harm of IVP, reported high prevalence among women involved in transactional sex, and evidence of broader social, cultural and economic influences on IVP, the authors carried out a qualitative research study to explore IVP and investigate motivations for IVP among participants in an observational study in the Lake Victoria region of Tanzania and Uganda who have been found to have high HIV incidence and high prevalence of reported transactional sex. The aim of this paper is to describe the drivers and motivations for IVP amongst these women.

## Methodology

### Selection of participants

Qualitative data collection was nested within an observational cohort study conducted among women at increased risk of HIV in Tanzania and Uganda. The methods of the observational cohort study have been described elsewhere (Kapiga et al., 2013; Vandepitte et al., 2011). In brief, in Tanzania the cohort study recruited 966 HIV negative women employed in bars, guesthouses and other food and recreational facilities in three towns adjacent to gold or diamond mines in North–West Tanzania: Geita, Shinyanga, and Kahama. HIV incidence was 3.7 per 100 women-years (Kapiga et al., 2013). In Uganda, the cohort study recruited 645 HIV negative and 382 HIV positive women who were either self-identified commercial sex workers (CSW) or employed in entertainment facilities such as bars, nightclubs, and lodges in the capital city of Kampala. HIV incidence among the HIV-negative participants was 3.6 per 100 women-years (Vandepitte et al., 2013). For both cohort studies the women were followed up every three months; follow-up in Tanzania ended after 12 months, and in Uganda is ongoing.

**Fig. 1:**
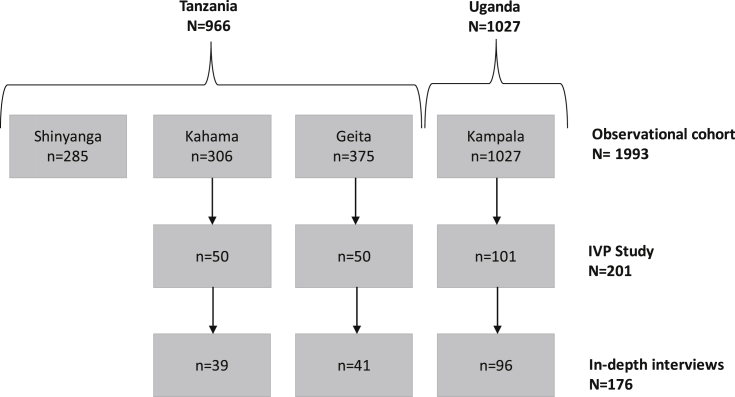
Flow chart of enrolment and numbers of in-depth interviews carried out in the IVP study.

Qualitative research was carried out as part of a sub-study to explore IVP in both countries (IVP Study). Consenting participants were asked to self-complete a pictorial paper-based diary every day for six weeks (42 days), and to participate in an in-depth interview (IDI) at the end of this period (see Francis et al., 2013; Francis et al., 2012). All participants were asked to only report on practices related to *intra*vaginal cleansing or insertion. In Tanzania, the IVP Study randomly selected 50 women from two of the three towns (Geita and Kahama, *N* = 681) at their enrolment visit. Of these, 39 and 41 completed IDIs respectively (see Fig. 1). For the IVP Study in Uganda, 101 women were enrolled by selecting every fourth participant at any follow up visit: nine were enrolled at their three month clinic visit, and the remainder between their 6–15 month visits. A total of 96 participants completed an IDI (see Fig. 1). In both countries, women who did not complete an IDI were those that were lost to follow-up. For the IVP study, data were collected from September 2008 to August 2009 in Tanzania and from June to September 2009 in Uganda. The quantitative data from the diary have been reported elsewhere, with a prevalence of intravaginal cleansing of 96% in Tanzania and 100% in Uganda, and a prevalence of intravaginal insertion of 10% in Tanzania and 46% in Uganda (Francis et al., 2013).

### In-depth interviews

IDIs obtained a detailed understanding of IVP reported in the diary and explored motivations for IVP use. Several topics were adapted from the WHO Gender, Sexuality, and Vaginal Practices (GSVP) study questionnaire, including questions on IVP initiation, traditional and modern influences, the nature and origin of products, physical results after using IVP, harmful effects from IVP, and if the participant's partner was aware of IVP use (Martin Hilber, Hull, et al., 2010). In addition, women were asked about their work, family life, sexual relationships, hygiene during menstruation, and general bathing habits. All IDIs were conducted by female research assistants in a private room at the cohort study clinics. In Tanzania, all IDIs were digitally recorded with the exception of one participant who did not consent to recording and instead notes were taken; and in Uganda, given the concerns over use of recording in this setting, notes were taken with direct quotations noted. The interviewers were experienced with this approach.

### Focus group discussions

At the end of the study, eight FGDs were carried out in Tanzania with female traditional healers (2), male bar patrons (2), main cohort participants who reported intravaginal insertion (2), and main cohort participants who reported intravaginal cleansing (2). Each FGD included 8 to 10 participants, and main cohort participants were selected irrespective of whether they were in the IVP study. The FGDs were led by same-sex research assistants and took place in a private place in a local bar or restaurant during closed hours. Topics included prescribing with IVP (female traditional healers); male expectations of the vaginal state and knowledge of IVP (male bar patrons); and motives for IVP, types of IVP, stigma related to IVP and resistance or support for changing IVP (women who reported cleansing and insertion). All topic guides included questions on cultural and social norms surrounding IVP and sexual relationships.

### Analysis

In Tanzania, all FGDs and IDIs were conducted in Swahili, recorded, transcribed and translated into English. An external translator quality checked 20% of the translations. In Uganda, all FGDs and IDIs were conducted in English or Luganda, and notes were written in English. In Uganda, the IDIs were written up from notes immediately after each interview. Coding and analysis of the translated transcripts and notes of FGDs and IDIs were carried out using NVIVO version 8 (QSR International Pty Ltd., Doncaster, Australia). Demographic details from the IDI participants' clinical interviews were imported from the main cohort study database to NVIVO.

The data analysis was guided by a framework analysis approach (Pope, Ziebland, & Mays, 2000). An initial coding structure based on the study objectives was developed *a priori* by authors SF and SL. The social scientists coordinating the qualitative research at the two sites (FZ and BA) were trained in NVIVO and simultaneously carried out initial coding on 10% of the transcripts before checking with each other for consistency. Following this, the coding structure was revised by SL, SF, FZ, and BA according to concepts emerging from the data. On completion of coding, SL conducted a comparative analysis across the concepts to explore themes related to motivations and drivers of IVP.

### Ethical statement

The main cohort studies and the qualitative sub-study were approved by the London School of Hygiene and Tropical Medicine Ethics Committee, the Tanzanian National Institute for Medical Research Medical Research Coordinating Committee, the Science and Ethics Committee of the Ugandan Virus Research Institute and the Ugandan National Council for Science and Technology. In both sites, participants were required to give written informed consent.

## Results

### Tanzanian participants

Demographic data from the IDI participants' clinical interview are reported in Table 1. In Tanzania, women attending the IDI had a mean age of 28. About half of the women had completed primary school, and a few reported never attending school. The majority of the women were Christian with the remainder being Muslim. Just over half of the women worked in bars, hotels and guesthouses, and the other half worked in other facilities such as independent food vendors (*mamalishe*) or small grocery stores. Age of first sex ranged from 13 to 24. Most women reported being separated, divorced or single, and relatively few women reported living with a partner. In Tanzania, 41% of the participants reported receiving money or gifts for sex in the past three months.

Further characteristics were obtained from the IDI data. Around half the women reported that they earned less than 1 US dollar a day. No data were obtained for the money earned or types of gifts given for transactional sex. The majority of women lived alone in a rented single room or in a compound at their place of work. *Mamalishe* were more likely to live with their family (partner and/or children), although the majority of women reported leaving their children with a relative in their home town or village to enable them to work. Most women (71) reported at least one sexual partner. Of these, 32 women had only regular partners (defined as more than one year duration), two had both regular and casual partners, and 37 had only casual partners. Of those that reported a partner, 23% reported more than one sexual partner. Four of the women reported having experienced intimate partner violence from a previous partner.

### Ugandan participants

Demographic data from the IDI participant's clinical interview reported in Table 1 showed that the women had a mean age of 28 years. Half the women never went to school or never completed primary school. Most women were Christian. Most were separated; divorced or single, and only 11% were cohabitating with a partner. The age of first sex ranged from 8 to 21. Sixty-one per cent of women were self-identified CSW (data not shown), and 93% stated that they had received money or gifts for sex in the past three months.

Data from the IDI showed that most of the women lived in rented single rooms in the slums. Most women (86%) had children, and 19% of the women reported that they had terminated a pregnancy at least once. Of the 92 women who reported a sexual partnership: 25 had regular partners, 22 had casual partners, and 45 had both regular and casual partners. Very few women (12%) reported that their regular partners were aware of their involvement in sex work. The money women earned from sex work ranged from around US$1.5 to US$9 per client. Nearly half (43%) of the women reported having experienced both physical and psychological violence perpetrated either by their partners or male sexual clients. As well as physical violence women reported being forced to have unprotected sex.

### General description of IVP

Intravaginal cleansing was reported by all women who attended an IDI, and it was seen as a normative practice routed in ethnic and religious tradition (both Christian and Muslim). In the IDIs, participants reported cleansing daily at different times: during bathing; after toileting; and before and after sex. The most common type of cleansing was internal washing with a finger and water. Whilst the use of water was desirable, none of the women had access to running water in their homes and instead sourced water from a communal well or tap, or a water vendor. When they were unable to access water, they used a piece of dry cloth or toilet paper. Around half of the women also used soap with water. A variety of soaps were used including body washing soap, antiseptic soap, or powdered or solid bars of laundry soap. They emphasised that soap ‘kills germs’:

‘… there is one type of soap that I use called *Kaisiki*; it is medicated, it gives a smell. I don't trust other types of soap. It actually says on the wrapper that you wash inside the vagina with that soap, you will remove the bad smell; it will kill the germs.’ (Cleansing FGD, Geita, Tanzania)

Some women reported negative effects from using soap including itching, vaginal infection, or a ‘loose and soft’ vagina which stopped them using soap. Others stopped following advice from a health professional, including the cohort study clinical staff. In FGDs, when asked if they would stop using soap if they were told if may increase their risk of HIV, all of the Cleansing and Insertion FGD participants agreed that they would.

‘If you actually tell me that if I use soap I can get any adverse effects, I can't continue using soap; I will have to wash inside the vagina with water only.’ (Cleansing FGD, Geita, Tanzania)

Most of the women were introduced to cleansing during adolescence by a female relative, usually their mother, aunt, sister, or grandmother. Most commonly this was when they started their menses or were given instruction about sex or marriage:

‘My mother told me that a woman was supposed to be clean all the time; it was when I had my first menses. She showed me how to use the middle finger to cleanse inside the vagina.’ (21 year old CSW, IDI, Kampala, Uganda)

‘[My mother] told me, “once you get married you shouldn't be dirty, when you have sex with your husband you must bathe and you must clean inside the vagina.”’ (43 year old Guesthouse worker, IDI, Geita, Tanzania)

Other women reported that they were introduced to cleansing in their twenties by peers. In particular, many of the Ugandan CSW learnt new cleansing practices from other CSW. A few women also learnt about cleansing through public health messages, either at school or in health centres:

‘It is due to biology at school, I mean according to how they taught us, how to do cleanliness.’ (20 year old Bar worker, IDI, Geita, Tanzania)

Insertion was less commonly practiced. In IDIs, only nine Tanzanian women and 37 Ugandan women reported inserting a commercial or traditional substance in the previous six weeks. The low frequency of reported insertion in Tanzania may have been due to the stigma that women attach to the practice, and FGDs with the traditional healers in Tanzania suggested that it is far more widely practiced than reported by these women. Insertion was often taught as part of initiation into womanhood, by traditional healers, or by *Ssenga* (literally paternal aunt in Luganda). However, in Kampala women seeking solutions to physical, sexual or emotional problems also learnt about insertion practices from female peers or through hearsay. The higher frequency of the practice among the Ugandan women also suggests that it was linked to the demands of commercial sex work (see below).

Women who reported insertion and traditional healers who supplied medicine (in Swahili, *dawa*) reported several different substances used. The traditional healers in Tanzania described the use of pulverised herbs, leaves and roots. As well as traditional *dawa*, Tanzanian women reported inserting snuff (pulverised tobacco), lemon leaves, cassava leaves, lemon juice, beer, *Konyagi* (a Tanzanian spirit similar to gin), and *shabu* (locally mined alum used to purify water). Substances reported by Ugandan women included traditional herbs, Coca-Cola, Omo (laundry detergent), honey, salt, beer or Vaseline. Women either inserted the substance directly in the vagina with a finger, or mixed with ghee (clarified butter) or Vaseline and inserted with a finger. Substances were inserted by the women when experiencing pain, in preparation for sex, or when prescribed by a traditional healer for sexual, relationship or health purposes. Such substances were reported to directly alter the vaginal state (e.g. tightness, removal of fluids), or to alter the behaviour of the sexual partner when contact was made during sexual intercourse (as described below).

Women reported that they were willing to stop insertion of commercial substances, particularly when hearing negative stories from other women, or from their own negative experiences:

‘And there is a medicine, I was given last year, I mixed it with snuff but it irritated me very much, and that is what made me stop inserting medicine inside the vagina.’ (36 year old Guesthouse worker, IDI, Geita, Tanzania)

Whilst many IVP, such as cleansing, were normative, they were highly secretive and women rarely discussed their own personal practices, even with intimate partners.

‘He doesn't know, I mean he has never discussed whether I have cleansed inside my vagina.’ (19 year old Guesthouse worker, IDI, Kahama, Tanzania).

FGDs with the Tanzanian male bar patrons revealed that whilst none had discussed IVP with their partners, men were aware of IVP. They suspected that women carry out IVP, particularly cleansing and insertion of substances, to secure and maintain sexual relationships. This secrecy was maintained by traditional healers who emphasised that the herbs they provide for insertion in the vagina were not detectable by men, and these herbs were effective because of the secrecy. The secrecy surrounding IVP often led to suspicion and distrust between women and men, especially if the women's post-sexual cleansing implied that the man was dirty or infectious:

‘…he challenged me as to why I was washing after having sex. I told him that I feel that my whole body smells, and I feel drier when I bathe and sleep comfortably. He said it is impossible for me to be bathe [at night] … he felt as if I am stigmatizing him or perhaps that I will leave him at any time.’ (39 year old *Mamalishe*, IDI, Geita, Tanzania)

### Motivations as embodiment of cultural and social norms and subjective well being

**Fig. 2:**
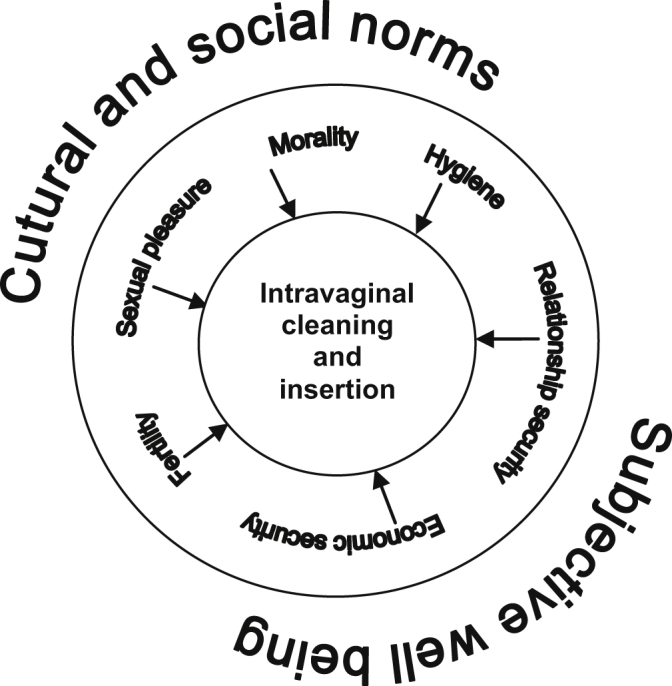
Conceptual diagram of the motivations for intravaginal practices among women who engage in transactional sex in an observational research study in Tanzania and Uganda. IVP were influenced by several overlapping motives including hygiene, morality, sexual pleasure, fertility, relationships and economic security. These motivations were strongly influenced by two factors: cultural and social norms and subjective well-being

Adapted from Martin Hilber et al., 2012.

During the IDIs, women revealed that IVP were influenced by a number of overlapping motives including hygiene, morality, sexual pleasure, fertility, relationship security, and economic security; thus, one type of IVP may serve more than one motivation at the same time. Additionally, these motivations were strongly influenced by two drivers: cultural and social norms and subjective well-being (see Fig. 2, adapted from Martin Hilber et al. 2012). In this way, IVP embodied norms of womanhood surrounding their moral, sexual, and reproductive roles in society. At the same time, women using IVP were attending to their personal emotional, economic, and physical state, including concerns with the transactional aspect of sex and HIV infection. Below, each motivation is shown to simultaneously address both drivers.

#### Hygiene

For both the Tanzanian and Ugandan women the main purpose of vaginal cleansing and the insertion of substances was to remove dirt (Swahili - *Uchafu* and Luganda *– Obukyafu)*. ‘Dirt’ refers to menstrual blood, vaginal discharge and fluids (in Swahili, *majimaji*) produced during sex, including vaginal fluids, semen or lubrication from condoms:

‘That dirt, even that of the condom, remains there [after sex] so I have to remove it.’ (20 year old Bar worker, IDI, Geita, Tanzania)

Hygiene was also important to women's own sense of well-being, to treat vaginal infection or discomfort or prevent HIV infection. Some women also inserted herbs to treat vaginal symptoms:

‘I use soap to clean inside the vagina in order to protect myself against HIV infection.’ (23 year old Guesthouse worker, IDI, Kahama, Tanzania)

‘When I started itching my aunt picked herbs for me and she told me to boil them with water and cleanse afterwards when the solution was still warm, then I got better.’ (22 year old CSW, IDI, Kampala, Uganda)

#### Morality

Both women and men participants emphasised that the ideal state of the vagina is “virgin-like” (in Swahili, *kama bikra)*. This state embodies women's physical and moral cleanliness:

‘The vagina should tighten so that the man may not discover… how many men she has [just] had sex with. She makes it narrow, she pulls it, she washes with cold water and it returns to its normal condition.’ (Cleansing FGD, Geita, Tanzania)

‘…if you insert herbs for tightening, it will be very hard for your partner to tell that you have just slept with another man.’ (27 year old CSW, IDI, Kampala, Uganda)

Ugandan women especially reported the use of commercial substances to enhance this virgin-like state. This included inserting *shabu*, Coca-Cola, or detergents used for washing clothes:

‘For instance there is a white powder [*shabu*] that women insert; it makes the vagina very tight.’ (35 year old CSW, IDI, Kampala, Uganda)

The FGD with Tanzanian male bar patrons highlighted the social pressures on women to ensure a virgin-like state, which the men described as lack of excessive fluid but not too dry, and free from smell and dirt. Smell was a particularly important concern for the men, which they associated with immorality:

‘Once you notice a smell you cannot do that [sex] act with excitement, you will do it whilst angry. Whether you paid for that or your partner decided to give you for free, if you find her dirty you will go ahead and do it two times or just once and then you leave for other activities.’ (Male bar patron FGD, Kahama, Tanzania)

Social moral norms of womanhood were strongly apparent, and women internalised these by reporting their own personal feelings of disgust and shame, and how this affected their perception of well-being:

‘You can have sex and feel that you are dirty; first of all you feel ashamed; you feel, I don't know, as if you belong to another world; you feel as if you are dirty.’ (26 year old Guesthouse worker, IDI, Geita, Tanzania)

#### Sexual pleasure

IVP were also conducted to enhance sexual pleasure. Teachings from elder women (or peers) emphasised to these women the importance of cleansing and tightening their vaginas to ensure pleasure for the men. There was, however, an emphasis by many women that cleanliness also enhanced their own sexual pleasure, and this was important for their well-being:

‘…when I clean inside the vagina, definitely I feel a change. …I mean something like a certain excitement.’ (22 year old *Mamalishe*, IDI, Geita, Tanzania)

‘That pleasure of being able to receive him…if I have washed inside the vagina in preparation to receive my husband, I feel pleasurable.’ (31 year old Guesthouse worker, IDI, Kahama, Tanzania)

Some women also reported inserting snuff or traditional herbs to enhance their own sexual pleasure. Although one other woman noted that:

‘You know this snuff, some people feel sexual desire [but] when you insert it, even if you had planned to have sex, the desire diminishes.’ (36 year old Guesthouse worker, IDI, Geita, Tanzania)

#### Treatment for fertility and pelvic pain

Fertility was discussed as an important aspect of womanhood, and women's concerns about pain in relation to their sexual and reproductive organs were important motivators for IVP. In Tanzania, *mchango* refers to lower abdominal cramp, which may or may not be menstrual. Several women were advised to treat *mchango* by inserting snuff into the vagina every day until the pain eased. In Uganda, women reported inserting Coca-Cola to treat bruises and sores resulting from rough sexual intercourse. Other women reported inserting commercially available gels before sex to lubricate the vagina in order to reduce painful sex:

‘…naturally I don't have vaginal fluid, what I do is use the gel to smooth the vagina and to prevent the pain that I might get when having sex.’ (34 year old, CSW, IDI, Kampala, Uganda)

These practices were the most secretive and were strongly rooted in tradition. In Tanzania, traditional healers provide herbs for *mchango*, which was seen to prevent conception or cause infertility. To treat *mchango* traditional healers provided women with snuff mixed with ghee to insert into the vagina or herbs for ingestion. The traditional healers also treat infertility by removing evil spirits (in Swahili, *majini)* who prevent conception. Islamic healers remove *majini* by reading a specific text from the Quran or non-Islamic healers use drumming (in Swahili, *kupunga)* to drive out the spirits.

#### Economic security

Transactional sex, whether through casual encounters or commercial sex work, was an important means of securing income for these women and contributed to their financial well-being. Most of the women were aware that such sexual encounters put them at risk of HIV, and cleansed after sex to minimise their risk of infection.

Whilst men were aware that women involved in transactional or commercial sex have other sexual partners, normative expectations of a clean and chaste woman influenced their desires. The Ugandan CSW and some Ugandan and Tanzanian facility workers were explicit in discussing the importance of ‘being desirable’ as a way to attract clients to make money. This involves ensuring cleanliness and the virgin-like state, as discussed above:

‘…if you are going to have sex with a man you are supposed to cleanse, you go there when you are clean, it [the vagina] becomes attractive’ (27 year old Bar worker, IDI, Geita, Tanzania).

As well as cleansing to remove dirt and insertion to tighten the vagina, women reported using herbs or commercial substances to stop the flow of menses before transactional sex, as men were unwilling to have sex with menstruating women:

“One day, my friend came to pick me to go to the street and I told her I was menstruating and she told me to insert soda [aerated drink] to stop the blood. I did it and no blood came out; I had sex, got money and went back home.” (31 year old CSW, IDI, Kampala, Uganda)

Herbs are also provided by traditional healers for insertion to enhance a women's attractiveness. As one Uganda sex worker said ‘You may at times look unpleasant to the customers but if you use these herbs, they will become attracted to you.’ (34 years old CSW, IDI, Kampala, Uganda)

#### Relationship security

Whilst economic needs drove many of the women's relationships with men, women also talked their desire for supportive and loving relationships. Such relationships were reported as important for their well-being. However, the means to secure these relationships were often derived through traditional practices, which drew on ideas of women's sexual power over men. Such traditional practices, prescribed by traditional healers, involve the insertion of herbs into the vagina with the aim of affecting the man during sexual intercourse. The desired effect is to secure a relationship with a new partner, to ensure love and fidelity, or to reduce discord in on-going relationships. For example, one Ugandan woman reported that her grandmother taught her to prepare a herbal mixture to ensure that her husband remained faithful and stop beating her:

‘I only insert it when I am going to have sex with him…I insert the herbs and have sex when the herbs are still there and thereafter, I remove the herbs and mix them in his food…and we have now settled our arguments after giving him this love potion.’ (22 year old CSW, IDI, Kampala, Uganda)

## Discussion

This study has reported qualitative findings on intravaginal cleansing and insertion practices among women living in urban and peri-urban environments involved in transactional sex, either as their main source of income or as a supplement to other sources of income, and represent a continuum of transactional sex (MacPherson et al., 2012). We aimed to describe, IVP and motivations for use in this population. Findings from this study revealed that IVP were influenced by a number of overlapping motives including hygiene, morality, sexual pleasure, fertility, relationship security and economic security. These are similar motivations reported by women in comparable contexts (Allen et al., 2010; Bagnol & Mariano, 2008; Gafos et al., 2010; Mann et al., 1988; Martin Hilber, Francis, et al., 2010; Martin Hilber, Hull, et al., 2010; Martin Hilber et al., 2012; Scorgie et al., 2009; Turner et al., 2010). In this study, these motivations were driven by adherence to cultural and social norms surrounding womanhood, but also attendance to personal well-being, especially in relation to sexual health, sexuality, and relationships.

Diary reports from our study revealed that intravaginal cleansing was highly prevalent amongst these women (96% in Tanzania, 100% in Uganda) (Francis et al., 2013). Interestingly, there were differences *within* this study population by country: Ugandan women had a higher prevalence of insertion (46% versus 10%) and used more commercial products (Francis et al., 2013). There were two likely explanations for these differences: participants from Kampala were in an urban environment with more access to commercial products; and a higher proportion of women in Uganda reported commercial and transactional sex and higher numbers of lifetime sexual partners. Data from the diaries have shown that frequency of cleansing and prevalence of insertion were related to frequency of sex in this population (Francis et al., 2013).

### Understanding IVP in the context of transactional sex

A particularly strong motivation for IVP amongst study participants was to maintain sexual health and conform to the social norms of a respectable woman. Participants used intravaginal cleansing practices introduced in their adolescence by female family members to reduce their risk of disease, particularly reproductive tract infections and HIV, which they perceived to be enhanced by the presence of ‘dirt’ in their vaginas. They described any substance that was ‘out of place’ in the vagina as dirty (see Douglas, 2002). These ‘out of place’ substances include semen, menstrual blood, or excessive fluid (i.e. wetness). The desire to remove these ‘dirty’ substances has also been noted with women involved in transactional sex in Mwanza City (Allen et al., 2010), and by women in other contexts in sub-Saharan Africa (see Beckmann, 2010; Braunstein & van der Wijgert, 2003; Leclerc-Madlala, 2002). Beckman (2010) suggests that for Zanzibari women, fluids linked with reproduction become ‘dangerous and polluting’ when they are of no further use (p. 628). Amongst the participants in this study, including male bar patrons from FGDs, vaginal wetness also indicates moral ‘dirtiness’, indicating that a woman is unfaithful or promiscuous. Leclerc-Madalla (2002) also found that amongst the Zulu ‘associations of “wet” and “loose” vaginas are mirrored in local discourses on morally “loose” women’ (p. 14). Thus, the dirt removing practices of the women in our study, either through cleansing internally with finger, cloth, water with or without soap, or insertion of cleansing products, enabled them to present themselves as physically and morally clean, and fulfil social and cultural norms surrounding womanhood in these contexts. Women's concerns with fertility were also a motivation for IVP, as well as the reduction of pelvic pain.

Participants were also motivated to use IVP to address concerns about sexuality. Whilst IVP practices to tighten and present a virgin-like state were done to enhance male sexual pleasure, women also cleansed and inserted substances to enhance their own sexual pleasure. This contrasts with other studies in Mozambique and South Africa that suggest that women's motives for vaginal practices only focus on sexual pleasure for men (Braunstein & van der Wijgert, 2003; Scorgie et al., 2009). Bagnol and Mariano (2008) also note, however, that vaginal practices amongst Mozambican women affect sexual wellbeing, which was seen as ‘a view of having a pleasurable and successful sexual encounter’ (p. 583). For the women in Mozambique “success” was understood as the conception of a child (Bagnol & Mariano, 2008). For the women in our study, “success” was described as involving a complexity of desirability and sexual pleasure driven by social rules and economic needs. Ethnographic work with similar women in Mwanza City has revealed a rejection of traditional ideas of men's control over women's sexuality and a wish for sexual freedom, albeit within prevailing moral ideas of the respectable woman (Lees, 2013).

IVP also addressed women's concerns with securing and maintaining relationships. The use of substances in the vagina to manipulate human relations is not unique to the women of Tanzania and Uganda. Bagnol and Mariano (2008) reported similar ideas among women in Mozambique and suggested that their actions were ‘part of an ongoing effort to transform the world around them or to seek to ensure balance and harmony’ (p. 583). Scorgie et al. (2009) found that vaginal practices among Zulu women provided a means to take control of a relationship and ensure fidelity. In our study, an important aspect of manipulating human relations was to ensure attraction, love, and fidelity through the secret insertion of substances prescribed by a traditional healer. The use of “love potions” has been described in detail by Wickström (2008). Love potions for the Zulu ‘offer one way to “do love”, to strengthen a relationship or win somebody's love, which is both a possibility and a threat in people's lives' (Wickström, 2008, p. 47). For these women, it was trust and fidelity that women sought and the ensuing security of a relationship that IVP provided. Thus, certain IVP drew on traditional practices and a cosmological view that human relationships can be manipulated.

### Adaptation of IVP to reduce harm

In light of the growing evidence around the link between IVP and HIV risk, there are concerns within the HIV prevention research community that women may need to adapt some types of IVP, such as reducing the insertion of substances which may harm the vaginal epithelium (Martin Hilber, Chersich, van de Wijgert, Rees, & Temmerman, 2007). In a systematic review of meanings and motivation of vaginal practices, Martin Hilber et al. (2012) found that in a range of contexts women adapt IVP to their needs and circumstances. This supports our findings that women in Tanzania and Uganda have adapted their practices, particularly to address their subjective well-being: participants were concerned about their health, and therefore cleansed to avoid reproductive tract infections and HIV acquisition. The findings reveal that economic motives also provide a stimulus for adapting IVP in this population as women must attract and maintain sexual partners with the “preferred vaginal state”, whilst protecting themselves from infection.

Many women reported willingness to eliminate the use of soaps or other potentially harmful substances if advised by health workers; however, they may be more resistant to eliminating all types of IVP especially those associated with deep cultural and social norms of womanhood. The variety of different products to enhance sexuality and sexual health used between Tanzania and Uganda has provided further evidence that IVP could be adapted. Women chose products based on availability, but also by highly localised notions of effectiveness and peer recommendations (i.e. hearsay). In many cases this led to the use of substances that were never intended for IVP, such as snuff, Coca-Cola and *shabu*. However, women may be willing to replace a “harmful” practice with another type of IVP that addresses the same motivation. Importantly, interventions that address cultural and social norms, for example by collaborating with older experienced women or traditional healers, or the use of a peer educator programme or social marketing, may be able to provide further support for a modification of IVP in this population.

A limitation of this study was that IVP is a sensitive behaviour and therefore participant responses may have been subject to social desirability bias. This bias may have been magnified because the IDI took place within the study clinics where participants may have received messages from cohort clinic staff about avoiding potentially harmful vaginal practices, or that simply being in a study about IVP changed IVP behaviour, that is the Hawthorne effect (McCarney et al., 2007). This may have been especially so with insertion, which was rarely reported in Tanzania. It must also be noted that reports by traditional healers suggesting that insertion was more widespread in Tanzania may also be biased given that their livelihood depends in part on prescribing products for insertion.

## Conclusion

In this population, interventions aimed at modifying, reducing or eliminating IVP should attend to overarching cultural and social norms and economic drivers in addition to individual behaviour change and adherence. Interventions that encourage IVP cessation may bias reporting on IVP, and may not have a long-term robust effect. Alternatively, interventions that take account of these norms and drivers could encourage women to cleanse with plain water as an alternative to other potentially harmful substances, and engage key community members (e.g. experienced older women or traditional healers) to support the intervention. Additionally, in the context of microbicide intervention studies, women participating in a clinical trial could be encouraged to delay cleansing after sex (rather than eliminate cleansing) if microbicide efficacy depends on the product remaining in the vagina for a period of time after sex.

## Figures and Tables

**Table 1:** Characteristics of the Tanzanian and Ugandan IDI participants contributing to the qualitative data analysis.

	Tanzania (*n* = 80)	Uganda (*n* = 96)
Age (mean years, range)	29 (18–44)	28 (17–51)
*Highest level of education*		
Never went to school	7 (9%)	8 (8%)
Primary incomplete	15 (19%)	40 (42%)
Primary complete	41 (52%)	18 (19%)
Entered secondary (complete/incomplete)	16 (20%)	30 (31%)
*Religion*		
Christian	59 (75%)	75 (78%)
Muslim	20 (25%)	21 (22%)
*Marital status*		
Married	13 (15%)	5 (5%)
Separated/divorced	38 (48%)	67 (70%)
Widowed	4 (5%)	7 (7%)
Single	24 (30%)	17 (18%)
*Cohabitating with a partner*		
No	60 (76%)	89 (89%)
Yes	19 (24%)	11 (11%)
*Main employment*		
Commercial sex worker	0	58 (60%)
Bar/hotel/guesthouse worker	48 (60%)	30 (31%)
Other facility worker	31 (40%)	8 (8%)
*Transactional sex in the past 3 months*		
No	47 (60%)	6 (6%)
Yes	32 (41%)	90 (94%)
Age of first sex (mean years, range)	17 (13–24)	19 (8–21)
*Total lifetime partners*		
0–4	31 (39%)	3 (3%)
5 or more	26 (33%)	30 (31%)
Don't remember	22 (28%)	63 (66%)
*Ever forced to have sex*		
No	68 (86%)	55 (57%)
Yes	11 (14%)	41 (43%)
